# A novel prognostic factor TIPE2 inhibits cell proliferation and promotes apoptosis in pancreatic ductal adenocarcinoma (PDAC)

**DOI:** 10.7150/ijms.51497

**Published:** 2021-03-11

**Authors:** Yuqi Sun, Shougen Cao, Zequn Li, Xiaodong Liu, Jinxiang Xu, Yulong Tian, Shuai Shen, Yanbing Zhou

**Affiliations:** 1 Department of Gastrointestinal Surgery, The Affiliated Hospital of Qingdao University, Qingdao, Shandong, People's Republic of China.; 2 Department of Hepatology, The First People's Hospital of Luoyang City, Luoyang, Henan, People's Republic of China.; 3 Department of Anorectal Surgery, Weifang People's Hospital, Weifang, Shandong, People's Republic of China.

**Keywords:** TIPE2, PDAC, prognosis, proliferation, survivin

## Abstract

**Background:** Tumor necrosis factor (TNF)-alpha-induced protein 8-like 2 (TIPE2 or TNFAIP8L2) is a newly discovered negative immune regulator. Studies have shown that TIPE2 causes significant malignant biological effects and is differentially expressed in various malignant tumors. However, the expression and roles of TIPE2 in pancreatic ductal adenocarcinoma (PDAC) are largely unknown.

**Materials and Methods:** The expression of TIPE2 in PDAC tissues was assessed by immunohistochemistry, qPCR and western blot analysis and related clinicopathological parameters including survival time were analyzed. After overexpression of TIPE2, cell proliferation and apoptosis analysis were conducted, and the associated underlying molecular mechanism was also explored.

**Results:** In the present study, TIPE2 was upregulated in early PDAC tissues, and TIPE2 expression decreased as the tumor progressed (*P*<0.001). TIPE2 expression was negatively associated with tumor size, TNM stage and metastasis of lymph nodes. Furthermore, as an independent risk factor, TIPE2 could be used to predict the survival of patients with PDAC (*P*=0.035). TIPE2 overexpression significantly suppressed the viability, proliferation and induced apoptosis of PDAC cells by inhibiting survivin and increasing the activity of caspase3/7.

**Conclusions:** For the first time, this study demonstrated that TIPE2 is an independent prognostic factor in PDAC. TIPE2 inhibited the proliferation and induced apoptosis via regulating survivin/caspase3/7 signaling pathway. These results indicated that TIPE2 is a potential biomarker for predicting the prognosis of PDAC patients and plays a pivotal role in the progression of PDAC.

## Introduction

Pancreatic cancer is one of the most common malignant tumors of the digestive tract system 1, 2. According to data released by the American Association for Cancer Research (AACR) in 2020, pancreatic carcinoma mortality ranks fourth among all malignant tumors, and its 5-year relative survival rate is only 8% 3. Pancreatic cancer is classified into ductal adenocarcinoma, acinar cell carcinoma, small gland cancer, large eosinophilic granulosa cell carcinoma, and small cell carcinoma according to its pathological classification. Among pancreatic cancer, pancreatic ductal adenocarcinoma (PDAC) is the most common type that accounting for 85%-90% of pancreatic cancer 4. At present, the treatment of PDAC is mainly based on surgery combined with radiotherapy and chemotherapy 5, 6. However, most PDAC patients are found to be in an advanced stage and often have a poor prognosis due to the insidious onset 7. Therefore, discovering molecular markers of PDAC with high sensitivity and specificity are of great clinical significance to improve the prognosis for this devastating disease.

Tumor necrosis factor-a-induced protein 8-like 2 (TNFAIP8L2, TIPE2) was identified for the first time in experimental organ encephalomyelitis (EAE) mice 8. The protein sequence of TIPE2 was found to be highly homologous to the other TNFAIP family members such as TIPE, TIPE1 and TIPE3 9-11. TIPE2 gene is highly expressed in immune organs of mice but is not expressed in other normal mice tissues and organs 8, 12, 13. Subsequent studies have found that TIPE2 negatively regulates innate and adaptive immunity through Toll-like receptor (TLR) and T cell receptor (TCR) signaling pathways. Unlike mice, TIPE2 is widely expressed in human tissues 14. As key members of immune cells, peripheral blood mononuclear cells (PBMCs) including lymphocytes, monocytes and dendritic cells play a key role in immune activities. The aberrant expression of TIPE2 was found in PBMCs of patients with systemic lupus erythematosus (SLE) or chronic hepatitis B and asthmatic children 15-17. In addition, it has been found that TIPE2 may serve as an immunological negative regulator in the dynamic regulation of tumor immune balance 14, 18. However, the expression condition and roles of TIPE2 in PDAC remains largely unknown.

Caspase proteins are a group of structurally related cysteine proteases found in the cytosol 19. Caspases are activated by various signaling pathways to degrade or inactivate certain key cellular proteins that mediate apoptosis, and their expression are closely related to the morphological features of apoptosis 20, 21. Survivin belongs to the apoptosis inhibitory protein (IAP) family. It is one of the strongest inhibitor of apoptotic factors 22, 23. Survivin plays an important role in regulating cell mitosis 24. Previous study found that survivin expression in pancreatic cancer is higher than that in benign pancreatic tumors. The high expression of survivin in PDAC is accompanied by a decrease in the apoptotic index 25. It is currently believed that the inhibitory effects of survivin on apoptosis may be resulted from the direct or indirect interactions with caspases. Survivin stably binds to the terminal effector caspase-3 and caspase-7 in the form of homodimers, thereby inhibiting protein cleavage and blocking apoptosis 26, 27.

In the present study, we demonstrated for the first time that TIPE2 was highly expressed in early stage PDAC but its expression decreased in advanced PDAC. We also found that TIPE2 expression was closely related to lymph node metastasis and the prognosis of PDAC patients. By *in vitro* experiments, we demonstrated that TIPE2 may serve as a tumor suppressor by inhibiting proliferation and promoting apoptosis of pancreatic cells.

## Materials and methods

### Patients and tissue samples

Tumor specimens and related normal tissues from 74 patients with PDAC were collected at the Affiliated Hospital of Qingdao University. Clinical pathology classification and staging were determined according to the 8^th^ edition of the American Joint Committee on Cancer (AJCC) criteria 28. Detailed clinicopathological parameters, such as patient age, gender, tumor location, neurovascular invasion and lymph node metastasis, were recorded from Electronic Medical Record System (EMRS). The follow-up time ranged from 3 to 7 years. The primary endpoint was overall survival (OS), which was defined as the time from diagnosis until death or last follow-up. It is recommended that all patients with PDAC should be followed up regularly according to clinical guidelines after completing primary surgical treatment. In addition, samples of peripheral blood were obtained preoperatively, and tumor and the adjacent normal tissues were obtained postoperatively from 10 progressor and 10 non-progressor PDAC patients during surgery. All procedures were approved by the Ethics Committee of the Affiliated Hospital of Qingdao University. All patients provided informed consent.

### Cell culture and transfection

Human pancreatic ductal adenocarcinoma cell lines (PANC-1 and SUIT-2) were purchased from the Cell Bank, Type Culture Collection of the American Type Culture Collection (ATCC). These cells were cultured in DMEM (Gibco, CA, USA) containing 10% heat-inactivated fetal bovine serum (FBS; Invitrogen, USA) and 1% penicillin/streptomycin (Invitrogen, USA) in a cell incubator with 5% CO_2_ at 37 ^°^C. Furthermore, human PBMCs were isolated from the heparinized venous blood (5-10 mL) by Ficoll-Hypaque sedimentation (Lymphoflot, Bio-Rad, city, state if USA, country). The PBMCs were collected from the interphase layer, and the cells were washed two times with PBS and resuspended in RPMI 1640 (Gibco, CA, USA) supplemented with 10% FBS. PANC-1 and SUIT-2 cells were transfected with the PRK5-TIPE2 plasmid using Lipofectamine 2000 according to the manufacturer's protocols (Takara, Japan). YM-155 (Calbiochem, USA), a specific survivin inhibitor, was used in some experiments to inhibit the activity of survivin.

### Immunohistochemistry (IHC)

Immunohistochemistry was performed using paraffin-embedded tissue sections. Paraffin-embedded tissue sections were dewaxed and hydrated. Antigen retrieval was performed in a 0.01 mol/L citrate buffer solution (pH 6.0) that was heated to boiling for 2-3 min in a stainless-steel pressure cooker. Endogenous peroxidase was blocked with a 3% hydrogen peroxide solution for 20 min. Sections were blocked with 10% goat serum (Invitrogen, Carlsbad, CA, USA) for 15 min and then immunostained with a rabbit antibody against TIPE2 (15940-1-AP, 1:100, Proteintech, China) at 4 ^°^C overnight. Secondary staining was performed with antirabbit immunoglobulin G for 1 h at 37 °C using a MaxVsion kit (Toyobo, Japan) and 3, 3-diaminobenzidine peroxidase substrate kit (Maixin Co, China). Sections were counterstained with hematoxylin for 5 min at room temperature.

### Evaluation of immunohistochemical staining

Immunohistochemical staining was independently and blindly assessed by two experienced pathologists. When there were large discrepancies (score difference > 2), a third pathologist analyzed the results. Under a light microscope (magnification, ×100 and ×200), three fields of view were randomly selected for each sample, and each group of cells contained approximately 500 cells. Staining was semiquantitatively scored based on the hematoxylin staining intensity (0, negative; 1, weak; 2, moderate; and 3, strong) and the percentage of positively stained cells (0, 0%; 1, 1-25%; 2, 26-50%; 3, 51-75%; and 4, 76-100%). The two scores for each sample were combined to obtain the final score for TIPE2 expression (IHC sum score). The cut-off point for the definition of the expression level was as follows: 0-3, low expression; 4-8, high expression.

### Real-time quantitative PCR

Total RNA from transfected cells was extracted by TRIZOL reagent, and mRNA was reversely transcribed into cDNA using a Quantscript RT kit and SuperReal PreMix plus (TIANGEN, China). Glyceraldehyde 3-phosphate dehydrogenase (GAPDH) was used as the positive RT control, and the fold change was calculated by the following equation: 2^-ΔΔ^Ct. The sequences of the sense and antisense primers were as follows: GAPDH, 5'-AACGGATTGGTCGTATTGGG-3' and 5'-CCTGGAAGATGGTGAGGGAT-3'; TIPE2, 5'-CCGCCACGTTTGATCACT-3' and 5'-GCTTCCCTTCGTCTAGCAGC-3'; survivin, 5'‑GCACTTTCTTCGCAGTTTC‑3' and 5'‑GTGAGGTGTGCTGTTCGAGA‑3'.

### Western blot analysis

The expression of the relevant protein in the experiment was determined by western blot analysis. Cellular proteins were extracted using RIPA lysis buffer (Beyotime, China) containing a phosphatase inhibitor cocktail and phenylmethylsulfonyl fluoride (PMSF). Protein was then loaded onto a 12% SDS-PAGE gel and transferred to a PVDF membrane. After blocking with 5% skim milk, the membrane was incubated with a primary antibody at 4 °C overnight, and the membrane was then incubated with an HRP-conjugated secondary antibody. Bands containing proteins of interest were developed by the ECL chemiluminescence substrate (Beyotime, China).

### Cell viability assay

PANC-1 and SUIT-2 cells at the logarithmic growth phase were collected to prepare cell suspensions. According to the cell counting kit-8 (CCK8) standard curve, a total of 2000 PANC-1 cells and 3000 SUIT-2 cells were plated on 96-well plates. CCK8 analysis was used to determine the activity of inoculated cells at 0, 24, 48, 72, 96 and 120 h. Three replicate wells were set up for each time point, and the experiment was performed at least three times independently.

### Colony formation assay

PANC-1 and SUIT-2 cells were plated into a six-well plate at 500 cells/well, and the medium was changed every 3 days. After two weeks, cells were fixed with 10% formaldehyde and stained with 1% crystal violet. Colonies composed of more than 50 cells were counted under an optical microscope and calculated as a percentage of the control group.

### Flow cytometry for detection of apoptosis

PANC-1 and SUIT-2 cells in the logarithmic growth stage and in good growth condition were cultured overnight at 37 °C in DMEM medium to adjust the cell density to 3×105 cells/mL. Cells were collected and analyzed by flow cytometry according to the instructions of the AnnexinV-APC/7-AAD cell apoptosis kit.

### Caspase activity assay

Cells were trypsinized, collected and centrifuged at 4 °C for 5 min. The supernatant was removed, and 100 μL of lysis buffer was added. The pellet was resuspended and placed in an ice bath for 15 minutes. The lysate was centrifuged for 10-15 min at 12000 rpm and 4 °C. Ac-DEVD-pNA (2 mM) was then added, and samples were mixed. Samples were incubated at 37 °C for 60-120 minutes. When the color began to significantly change, the A405 wavelength was measured. Detection of the protein concentration in samples was performed by the Bradford method.

### Statistical analysis

The relationship between TIPE2 expression and the clinicopathological parameters were analyzed using χ^2^ and Fisher's exact tests. Quantitative data were expressed as the mean ± standard deviation. Paired and non-paired t-tests were used to determine the difference between two groups, and one-way ANOVA was used to determine the difference among multiple groups. The diagnostic value of TIPE2 in PDAC was evaluated using receiver operating characteristic (ROC) curve analysis. Survival rates were calculated using the Kaplan-Meier method. Univariate and multivariate survival analyses were performed using the Cox proportional hazard model to determine independent prognostic factors. All statistical analyses were performed using SPSS software (version 23.0; IBM Corp, Armonk, NY, USA), and *P*<0.05 was considered to indicate a statistically significant difference.

## Results

### TIPE2 protein expression is associated with tumor progression in PDAC

**Figure 1:**
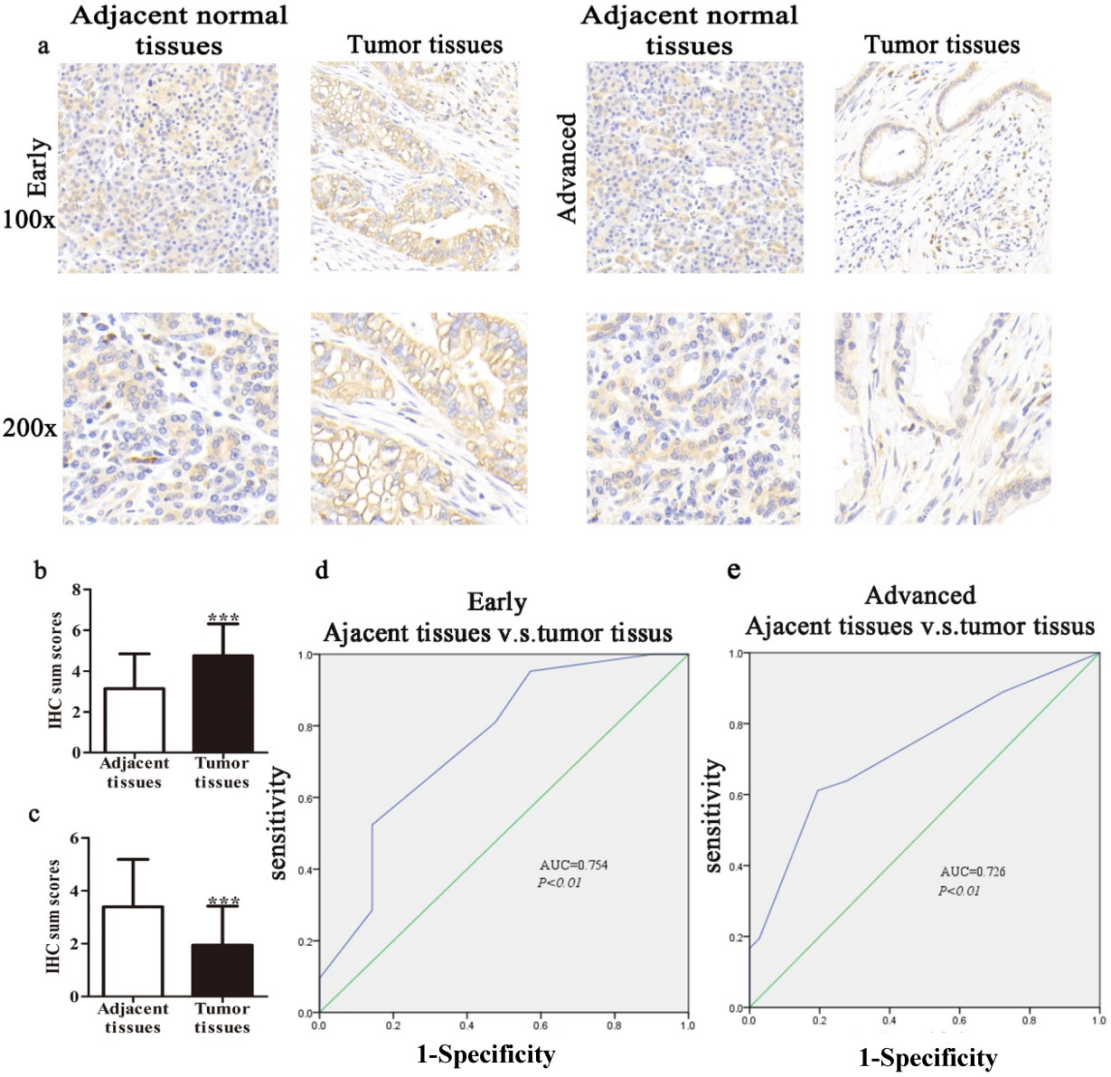
** TIPE2 expression and clinical significance in normal pancreatic tissues and early/advanced tumor tissues. a.** IHC results (×100 magnification and ×200 magnification) of TIPE2 expression in adjacent normal tissues and early and advanced PDAC tissues. **b.** TIPE2 expression was upregulated in early PDAC tissues compared to adjacent healthy tissue. ***P<0.001. **c.** TIPE2 expression was lower in advanced PDAC tissues compared to normal pancreatic tissue. ***P<0.001. **d.** The ROC curve shows clear separation between normal tissues and early PDAC tissues with an AUC of 0.754 (*P*<0.01). **e**. The ROC curve shows strong separation between adjacent normal tissues and advanced PDAC tissues with an AUC of 0.726 (*P*<0.01).

We collected 74 cases of PDAC tumor tissues and corresponding 57 cases of adjacent normal tissues to clarify the expression of TIPE2. IHC results showed that TIPE2 staining in early tumor tissues was significantly increased compared to that in adjacent normal tissues (*P*<0.001; Figs. 1a, b). However, the expression of TIPE2 was markedly decreased in advanced stages with tumor progression compared to that in normal tissues (*P*<0.001; Figs. 1a, c).

In our study, we constructed ROC curves to evaluate the diagnostic value of TIPE2 expression at different stages of PDAC. The AUC of the ROC curves showed that TIPE2 in discriminated PDAC tissues and normal tissues was up to 0.754 in the early stage (95% confidence interval (CI), 0.606-0.902; *P*=0.005; Fig. 1d) with an estimated sensitivity and specificity of 95.2% and 42.9%, respectively. Furthermore, the AUC for TIPE2 as a predictor for advanced stage was 0.726 in PDAC (95% CI, 0.608-0.844; *P*=0.001; Fig. 1e) with an estimated sensitivity and specificity of 61.1% and 80.6%, respectively.

**Figure 2:**
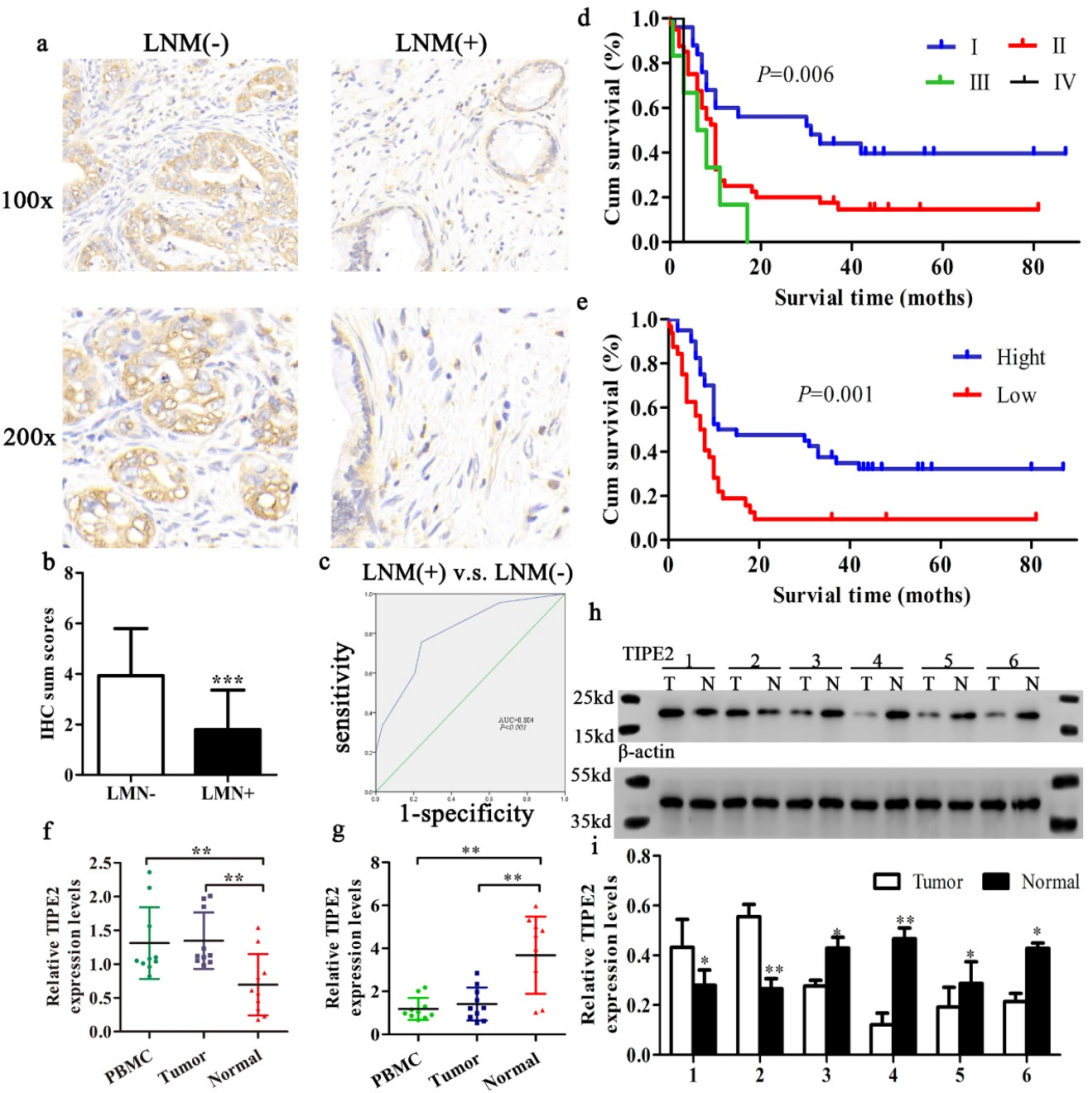
** Expression and significance of TIPE2 in tumor tissues with and without lymph node metastasis (LNM). Kaplan‑Meier survival curves of patients with PDAC. a.** IHC results (×100 magnification and ×200 magnification) of TIPE2 expression in PDAC tissues with and without LNM.** b.** Expression of TIPE2 was higher in PDAC tumor tissue without LNM compared to PDAC tumor tissue with LNM. ***P<0.001. **c.** The ROC curve shows separation between PDAC tumor tissues with and without LNM with an AUC of 0.804 (*P*<0.001). **d-e.** A log-rank test revealing the significant effect of tumor grade (**d**) and the level of TIPE2 (**e**) on the overall survival time of the patients.** f-g.** TIPE2 expression in PDAC tissues during progressor and non-progressor were measured by qPCR. **h-i.** The expression of TIPE2 in the adjacent normal tissues and PDAC tissues were detected by western blot analysis.

Furthermore, the expression level of TIPE2 mRNA was also confirmed in 10 progressor and 10 non-progressor pairs of PDAC tissue samples by qRT-PCR analyses. Consistent with the IHC results, TIPE2 was upregulated in tumor tissues and PBMCs compared to normal tissues in early stages of PDAC. However, the expression of TIPE2 mRNA was decreased in advanced cancer (*P*<0.01, Figs. 2f, g). Meanwhile, to further confirm whether TIPE2 was abnormally expressed in PDAC tissues, 6 cases of PDAC tissues and corresponding normal tissues (2 early stage, 4 advanced stage) were collected to detect the protein expression of TIPE2 by western blot (Figs. 1h, i).

### TIPE2 expression is closely related to the clinicopathological parameters of PDAC patients

To assess the clinical significance of TIPE2 expression in PDAC, the relationship between TIPE2 expression and various clinicopathological characteristics was analyzed (Table 1). The expression of TIPE2 was closely related to tumor stage (*P*<0.001), lymph node metastasis (*P*<0.001) and tumor diameter (*P*=0.030). By IHC staining, we also found that the TIPE2 levels decreased in tumor tissues with lymph node metastasis (LNM) compared to those in tumor tissues without LNM (*P*<0.001; Figs. 2a, b). To elucidate the relationship between TIPE2 and LNM, ROC curve was constructed. The AUC for TIPE2 as a predictor of LNM reached 0.804 (95% CI, 0.703-0.905; *P*<0.001; Fig. 2c) with an estimated sensitivity and specificity of 75.6% and 76.9%, respectively. However, TIPE2 expression was not associated with patient age, sex, tumor differentiation, location, neurovascular invasion and risk factors (*P*>0.05; Table 1). These data suggest that the decrease in TIPE2 levels may lead to tumor progression in PDAC and promote tumor proliferation.

### TIPE2 serve as an independent prognostic factor for patients with PDAC

This study followed patients with PDAC for 3-7 years after surgery with a median follow-up period of 4 years. The median survival time was 10 months (range from 1-87 months). The OS of the patients was 21.6%, of which the low and high TIPE2 expression of OS was 8.8% and 32.5%, respectively.

The Kaplan-Meier method was applied to evaluate the value of TIPE2 expression and prognosis in patients with PDAC. According to the survival curve, patients with low TIPE2 expression in samples had a shorter overall survival time than patients with high TIPE2 levels (Fig. 2e). In addition, according to the 8^th^ edition of AJCC cancer staging for pancreatic cancer 28, the TNM stage of the biopsy samples better reflected the prognosis of the patient (Fig. 2d). The Cox proportional hazards regression model was used to further explore the factors associated with prognosis. Univariate analysis suggested that tumor stage, tumor differentiation and TIPE2 levels were significantly associated with the OS of PDAC patients (Table 2). Subsequently, all the variables proved statistically significant in univariate analysis were included in multivariate Cox proportional hazards analysis. Multivariate analysis confirmed that tumor stage, tumor differentiation and TIPE2 expression were independent prognostic factors for PDAC patients (Table 2).

### Overexpression of TIPE2 suppresses the proliferation and promotes apoptosis of PDAC cells

The results described above indicated that TIPE2 expression was closely associated with clinicopathological features of PDAC. In addition, TIPE2 may contribute the tumorigenesis, progression in PDAC and TIPE2 expression was closely associated with the prognosis of PDAC patients. To further elucidate the role of TIPE2 in PDAC, we investigated the effect of TIPE2 on proliferation and apoptosis of PDAC cells.

**Figure 3:**
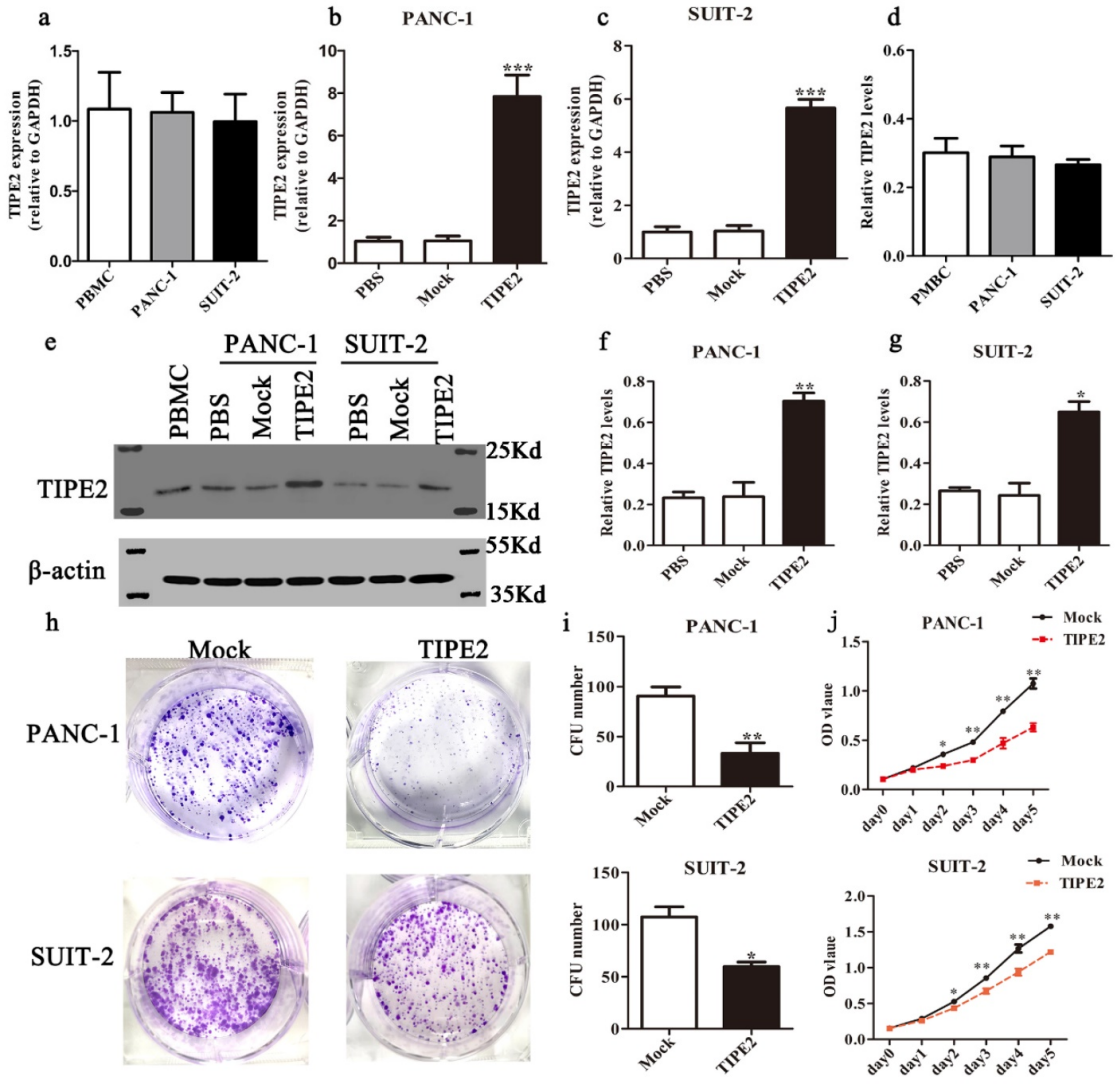
** TIPE2 overexpression suppresses the viability and proliferation of PDAC cells.** Real-time quantitative PCR (**a-c**) and western blot (**d-g**) analyses indicated that TIPE2 expression was markedly upregulated after transfection of the PRK5-TIPE2 plasmid in PANC-1 and SUIT-2 cells using PBMCs as a positive control. **h-j.** After TIPE2 overexpression, colony formation (**h-i**) and CCK8 (**j**) assays were conducted to evaluate the viability and proliferation of PANC-1 and SUIT-2 cells. Data represent the mean ± SD of three independent experiments. *P<0.05; **P<0.01; ***P<0.001.

**Figure 4:**
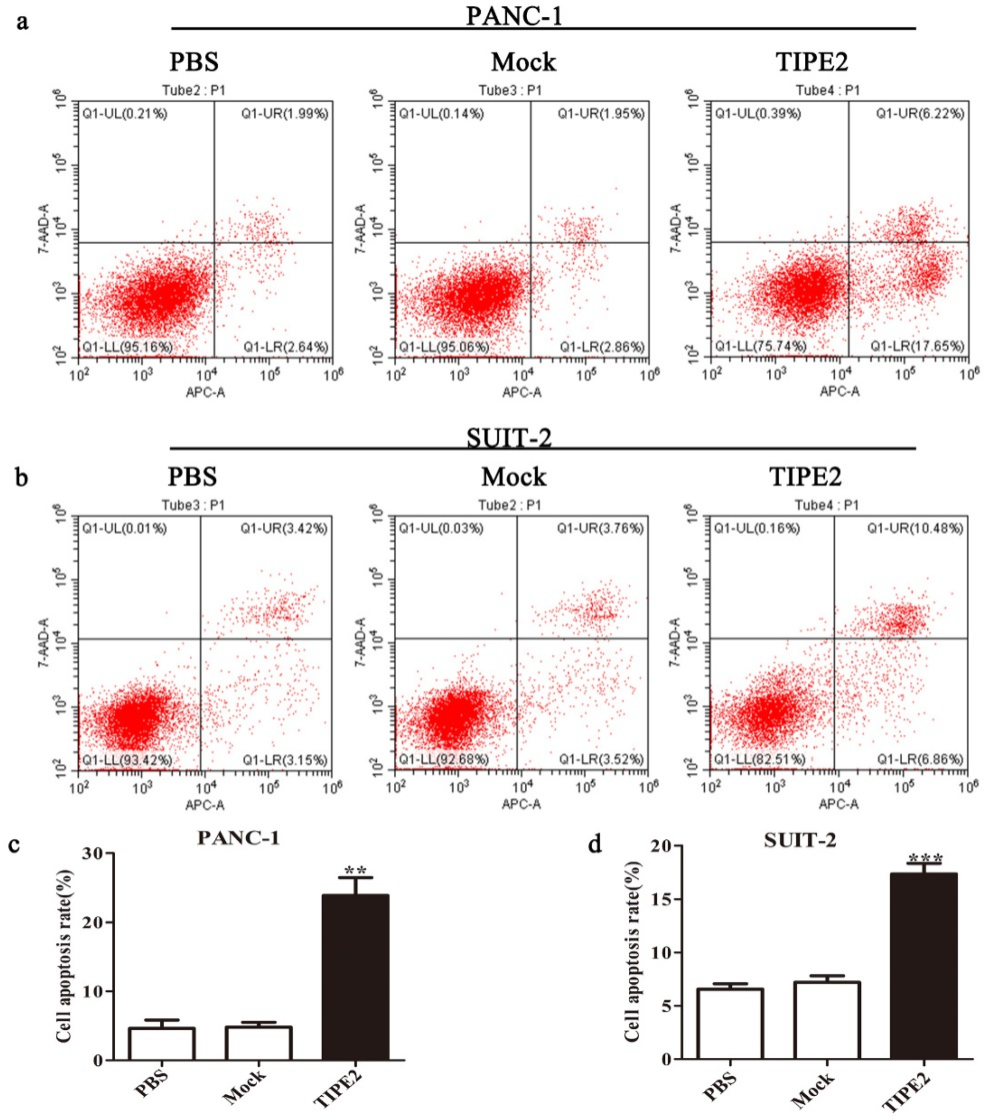
** Overexpression of TIPE2 promotes apoptosis of PDAC cells. a and c.** Cell apoptosis was detected by flow cytometry after transfection of the PRK5-TIPE2 plasmid into PANC-1 cells. **b and d.** Cell apoptosis was detected by flow cytometry after transfection for 48 h in SUIT-2 cells. Data represent the mean ± SD of three independent experiments. **P<0.01; ***P<0.001.

First, we detected the TIPE2 expression in PBMCs and the human PDAC cell lines PANC-1 and SUIT-2 by qPCR and western blot. No difference in TIPE2 expression between PBMCs, PANC-1 and SUIT-2 cells was shown (Figs. 3a, d). Meanwhile, it was discovered that TIPE2 expression was low in PBMCs, PANC-1 and SUIT-2 cells. Then TIPE2 was overexpressed in the two cell lines using the PRK5-TIPE2 recombinant plasmid. According to the qPCR and western blot results, TIPE2 was upregulated in the overexpression samples (Figs. 3b-g). In addition, CCK8 (Fig. 3j) and colony formation assays (Figs. 3h-i) were conducted to explore the effect of TIPE2 on cell proliferation. Overexpression of TIPE2 inhibited the proliferation of PDAC cells. Subsequently, we examined the effect of TIPE2 on apoptosis (Fig. 4). TIPE2 overexpression markedly induced apoptosis. Collectively, these results showed that TIPE2 inhibited tumor cell proliferation and promoted apoptosis in PDAC cells.

### TIPE2 promotes apoptosis of PDAC cells via targeting survivin protein

Our previous results showed that TIPE2 played a pivotal role in tumor progression. However, the detailed mechanisms remain unclear. Survivin, one of the most potent inhibitor of apoptosis, is a member of the IAP family 22, 29. Satoh 25 found that the expression rate of survivin in PDAC was 76.9%, and it played an important role in the progression of PDAC. Therefore, we speculated that TIPE2 might promote apoptosis by decreasing survivin in PDAC. To validate our hypothesis, we conducted the following experiments.

**Figure 5:**
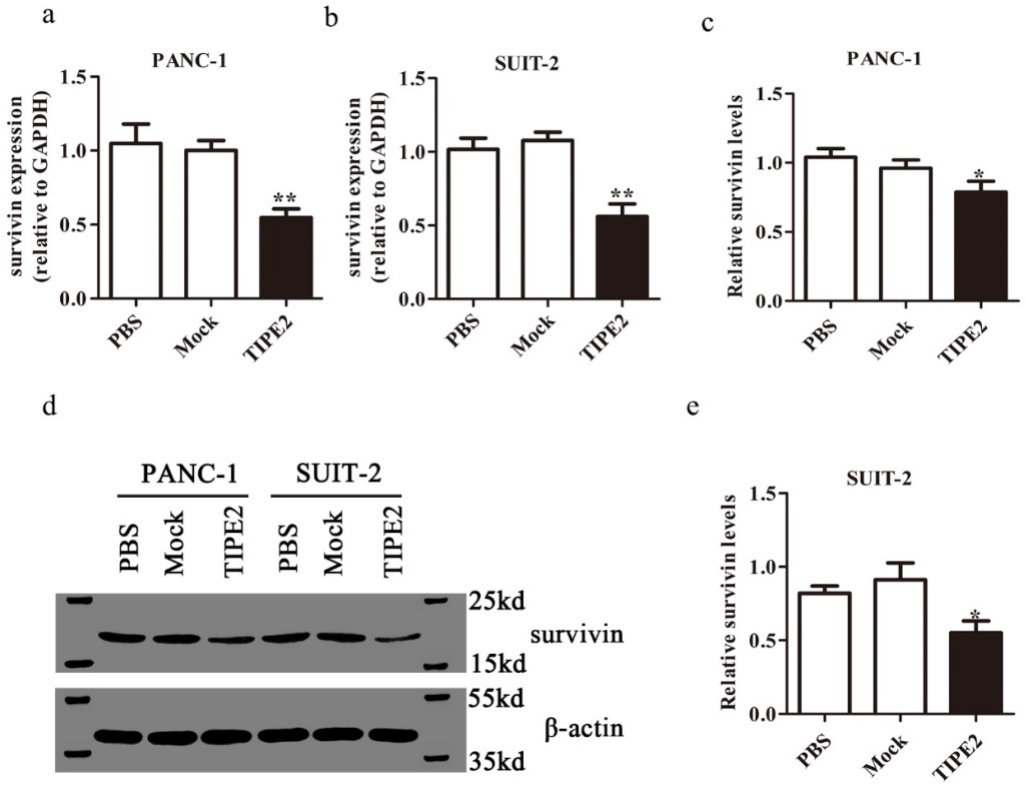
** Survivin expression after overexpression of TIPE2. a-b.** Real-time quantitative PCR showed that survivin expression was lower after transfection of the PRK5-TIPE2 plasmid into PANC-1 and SUIT-2 cells.** c-e.** The expression of survivin at the protein level was detected by western blot analysis after transfection for 48 h in cell lines.

**Figure 6:**
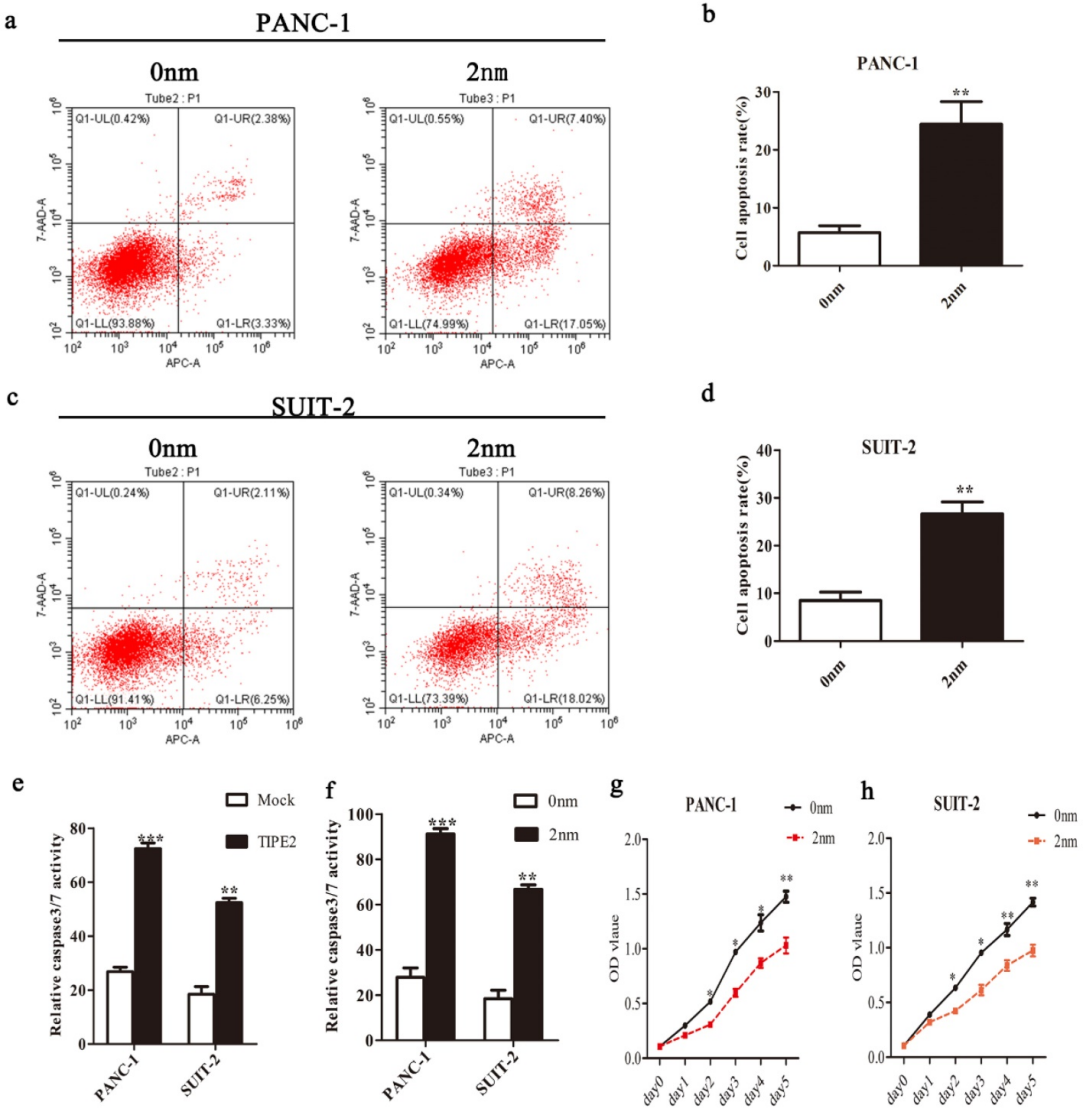
** TIPE2 promotes caspase 3/7 expression by inhibiting survivin in PDAC cells. a-d.** Cell apoptosis was detected by flow cytometry after transfection of the survivin inhibitor in PANC-1 (**a-b**) and SUIT-2 (**c-d**) cells. **e.** The activities of caspase 3/7 were increased after transfection of the PRK5-TIPE2 plasmid into PANC-1 and SUIT-2 cells. **f.** Survivin inhibitor increased the activities of caspase 3/7. **g-h.** CCK8 assays were conducted to evaluate the proliferation of PANC-1 and SUIT-2 cells after treatment of the survivin inhibitor.

We confirmed the high expression of survivin in PANC-1 and SUIT-2 cells at the mRNA and protein levels by qPCR and western blot, respectively (Fig. 5). Overexpressing TIPE2 with PRK5-TIPE2 in both cell lines significantly decreased the expression of survivin. Moreover, YM155 30, a survivin inhibitor, was used to investigate the effect of survivin on PDAC cell apoptosis. Inhibition of survivin activity effectively promoted PDAC cell apoptosis (Figs. 6a-d). Furthermore, by inhibiting survivin activity, we demonstrated that inhibition of survivin activity could suppress PDAC cell proliferation (Figs. 6g and h). Taken together, these results indicated that TIPE2 promoted apoptosis and suppressed proliferation of PDAC cells by inhibiting survivin.

### TIPE2 regulates the expression of survivin downstream effectors, caspase 3/7, in PDAC cells

It is well known that caspases are effectors of various apoptotic signals. It is currently believed that survivin inhibits the expression of caspase 3/7 to block apoptosis. Caspase 3/7 are important downstream effectors of survivin 26. Therefore, we investigated the effect of TIPE2 on the expression of caspase 3/7 in PDAC cells. The activities of caspase 3/7 after overexpression of TIPE2 and inhibition of survivin activity were detected (Figs.6 e and f). The results showed that TIPE2 induced the apoptosis of PDAC cells by inhibiting the expression of survivin and increasing the activities of caspase 3/7 subsequently.

## Discussion

In recent years, the incidence of PDAC has increased 2, 31. Despite the development of precision medicine and the increasing treatment strategies, the prognosis of PDAC patients is extremely poor due to the insidious onset of this disease 7, 32. Discovering novel biomarkers and treatment targets are of vital importance for improving the prognosis of PDAC patients. Although, TIPE2 mRNA expression level was not associated with overall survival of PDAC using the public prediction database. Nevertheless, the immunohistochemical results of this study showed the low expression of TIPE2 on the protein level could serve as an independent unfavorable prognostic factor in PDAC. As is well known that protein is the basic functional unit and protein level do not necessarily always correlate strongly with the mRNA level 33. Factors such as mRNA modification and transportation, protein degradation, and folding of the protein may also lead to inconsistency mRNA abundance and protein expression level 34. All these may account for the discrepancy between the mRNA level and protein level in predicting prognosis. Meanwhile, we demonstrated that TIPE2 expression is closely related to tumor proliferation and apoptosis.

At present, it is believed that there are various immunological surveillances in humans 35, 36. However, tumors progression may be closely related to their ability to escape immune surveillance and destroy immune homeostasis 37. The TIPE protein family is essential for maintaining immune homeostasis 11, 38. TIPE2, a member of the TIPE family, plays a negative regulatory role in the innate and adaptive immune responses 8, 14, 39. Research on colon cancer has shown that the level of TIPE2 in colon cancer tissues is significantly upregulated compared to adjacent normal tissues and that TIPE2 participates in the development of colon cancer by binding caspase 8, thereby inhibiting the TLR4 inflammatory pathway 40. Similarly, the expression of TIPE2 is also elevated in renal carcinoma, non-small cell lung cancer (NSCLC) and skin squamous cancer 41-44. In contrast, TIPE2 expression is decreased or even absent in some specific tumors, including primary liver cancer 45, gastric cancer 46, glioma 47 and prostate cancer 48. In the present study, TIPE2 expression was higher in early tumor tissues than in adjacent normal tissues, but TIPE2 expression decreased in advanced stage PDAC tissues. TIPE2 expression was negatively correlated with TNM stage and lymph node metastasis. These results may be related to the following two points. On one hand, TIPE2 may be selectively overexpressed in the glandular epithelium 45. In the early stage of PDAC, glandular metaplasia leads to the upregulation of TIPE2 expression. On the other hand, in the early stages of cancer, the body may stimulates antitumor immune responses, resulting in an increased TIPE2 level 49. With the development of tumors, the negative regulation of TIPE2 is gradually weakened and the expression of TIPE2 is decreased or even absent. This observation was also independently validated by qPCR and western blot. At the same time, PBMCs from PDAC patients was used as a positive control to ensure the accuracy of the research. In addition, the ROC curve showed that TIPE2 better distinguished lymph node metastasis. Moreover, TIPE2 expression was closely correlated with clinical prognosis and served as an independent prognostic factor. Taken together, these results indicated that TIPE2 may be an effective potential immunohistochemical marker for assessing the risk of tumor progression and predicting prognosis.

Previous studies have shown that TIPE2 acts as a tumor suppressor in tumors mainly by inhibiting tumor proliferation, migration, invasion and angiogenesis 18, 45. However, the role of TIPE2 in PDAC has not been elucidated. The results of this study showed that overexpression of TIPE2 significantly inhibited proliferation and induced apoptosis of PDAC cells. Therefore, our data indicated that although TIPE2 expression was upregulated in early PDAC tissues, TIPE2 was recognized as a tumor suppressor in PDAC.

To better elucidate the role of TIPE2 in inducing apoptosis, we examined the expression of an inhibitor of apoptosis, survivin, after TIPE2 overexpression. Survivin is not only the strongest inhibitor of apoptosis but also allows cells to escape from checkpoints, leading to abnormal cell proliferation in the G2/M phase of the cell cycle 24, 50. We demonstrated that TIPE2 inhibited the expression of survivin. Caspase 3/7 are the terminal effectors of apoptosis. In addition, caspase 3/7 have been shown to be key downstream factors for survivin 26, 51. Here, we demonstrated that TIPE2 regulated the activity of caspase3/7 in PDAC cells via survivin, to participate in tumor apoptosis and inhibit proliferation.

Similar to other solid tumors, the proliferation and apoptosis of PDAC cells are regulated by multiple genes. The occurrence and development of PDAC is a complex process involving multiple biological mechanisms and many signaling factors. The interconnection between these networks determines the proliferation and differentiation of malignant tumors. The regulatory effect of TIPE2 on survivin/caspase3/7 signaling pathways may be indirect. There may be other intermediate targets, involving more signaling pathways. In this study, we only investigated the roles and mechanism of TIPE2 in PDAC preliminarily. The more detailed molecular mechanisms remain to be further explored. In addition, we only collected pathological biopsies from 74 patients, which led to a lack of specificity in the ROC curve. A larger cohort needs to be constructed to further determine the precise predictive effect of TIPE2 in PDAC progression. Furthermore, our current research is limited to *in vitro* experiments, and *in vivo* experiments are also warranted.

## Conclusion

In the present study, we demonstrated for the first time that TIPE2 expression was upregulated in early tumor tissues but the levels of TIPE2 decreased in advanced stage PDAC tissues. TIPE2 expression was negatively associated with tumor size, LNM, and tumor TMN stage in PDAC. In addition, TIPE2 served as an immunohistochemical biomarker for predicting the prognosis of PDAC patients and assessing the degree of tumor malignancy. Mechanically, TIPE2 may act as a tumor suppressor by inhibiting survivin and regulating caspase 3/7. These results indicated that TIPE2 is a key regulator in PDAC and deserves further investigation.

## Figures and Tables

**Table 1:** Association of TIPE2 expression and clinicopathological characteristics in PDAC

Characteristic	Number	TIPE2 expression		*P* value
		Low	High	
**Number of patients**	74	33	41	
**Age (Years)**				
≤60	38	13	25	0.065
>60	36	20	16	
**Gender**				
Male	48	21	27	0.843
Female	26	12	14	
**Risk factor analysis**				
Smoke (yes/no)	50	10/10	10/20	0.239
Drink (yes/no)	49	9/11	11/19	0.556
Diabetes (yes/no)	50	6/14	5/25	0.265
**Tumor location**				
Head-neck	46	21	25	0.864
Body-tail	23	10	13	
**Primary tumor diameter (cm)**				
≤4	46	16	30	0.030*
>4	28	17	11	
**AJCC TNM stage^#^**				
I	26	2	24	<0.001*
II-IV	48	31	17	
**Lymph node metastasis**				
Negative	45	11	34	<0.001*
Positive	29	22	7	
**Tumor differentiation**				
I-II	49	24	25	0.288
II-III	25	9	16	
**Neurovascular invasion**				
Yes	32	15	17	0.730
No	42	18	24	

#The 8^th^ AJCC classification criteria.

**Table 2:** Univariate and multivariate Cox proportional hazard analyses of survival times of patients with PDAC

Variable	Univariate analysis			Multivariate analysis		
	HR	95%CI	*P-*value	HR	95%CI	*P*-value
TIPE2 expression	0.411	0.242-0.697	0.001*	0.495	0.257-0.951	0.035*
TNM stage	1.904	1.268-2.859	0.002*	2.036	1.059-3.913	0.033*
Neurovascular invasion	1.124	0.670-1.886	0.657	1.025	0.592-1.777	0.929
Tumor differentiation	1.986	1.158-3.406	0.013*	3.150	1.700-5.839	<0.001*
Age	1.581	0.943-2.652	0.082	1.215	0.698-2.114	0.491
Tumor diameter	1.115	0.976-1.274	0.110	0.882	0.712-1.094	0.253

HR, hazard ratio; CI, confidence interval.
